# Ulcerated Lesion of the Tongue as Manifestation of Systemic Coccidioidomycosis

**DOI:** 10.1155/2017/1489501

**Published:** 2017-03-13

**Authors:** Luis A. Mendez, Sergio A. Flores, Ricardo Martinez, Oslei Paes de Almeida

**Affiliations:** ^1^Basic Sciences, University of Montemorelos, School of Dentistry, Montemorelos, NL, Mexico; ^2^Oral and Maxillofacial Surgery, IMSS, Saltillo, COAH, Mexico; ^3^Oral Pathology, University of Montemorelos, School of Dentistry, Montemorelos, NL, Mexico; ^4^Department of Oral Pathology, University of Campinas, Piracicaba, SP, Brazil

## Abstract

Systemic mycoses and their oral manifestations are very rare. We present a case of a 60-year-old man with an ulcerated lesion on the lateral border of the tongue. Histologic studies revealed a granulomatous fungal infection by* Coccidioides immitis*. After pharmacological treatment, the lesion resolved. Recently, northern Mexico has been reported to be an endemic zone of* C*.* immitis* infections; therefore it should be considered in the differential diagnosis of mouth lesions. A comprehensive clinical history, physical exploration, and complementary studies are essential for an accurate diagnosis.

## 1. Background

Coccidioidomycosis was first discovered in Argentina in 1892 by a medical student with the observation of several patients developing dermatologic lesions throughout the body. Works developed by clinicians and scientists at Stanford University Medical Center were the main source of knowledge of this condition. Coccidioidomycosis can be acquired by inhalation of the organism or inoculation through the skin. Different terms were used when initially described such as San Joaquin fever, Desert fever, or Valley fever, which still remain used up to now [1]. The condition is endemic in the southwest USA, northern Mexico, and parts of Central and South America [2]. Usually* C*.* immitis* grows 5–30 cm under the ground, especially around burrows of rodents and reptiles. Risk factors for acquiring this infection include activities that expose the subject to contaminated dust such as archaeologist, military personnel, construction workers, hunters, earthquakes victims, and immunocompromised patients. It is more prevalent in man than woman. Skin manifestations of coccidioidomycosis are frequent, usually as papule, nodules, and verrucous lesions that may evolve to an ulcer or abscess. The most affected areas are the nasolabial groove and sternoclavicular area [3] Coccidioidomycosis is typically a granulomatous infection involving the lungs, rarely presenting in the mouth [4].

## 2. Case Presentation

**Figure 1:**
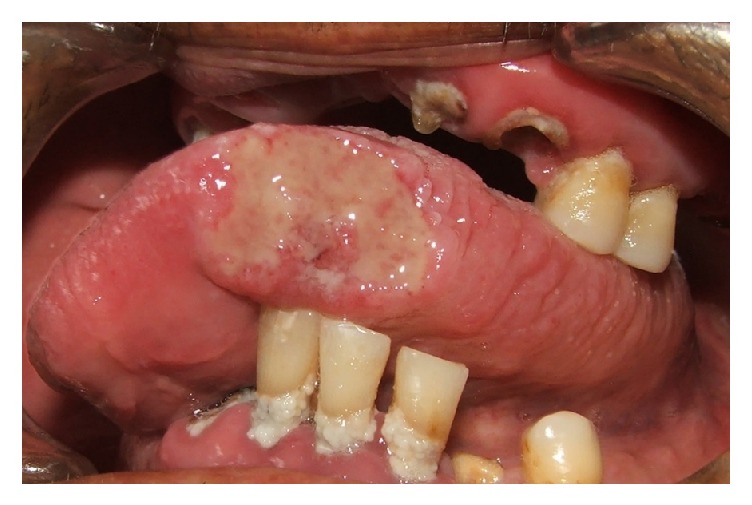
Lateral anterior border of the tongue showing a large ulcerated lesion with indurated borders.

**Figure 2:**
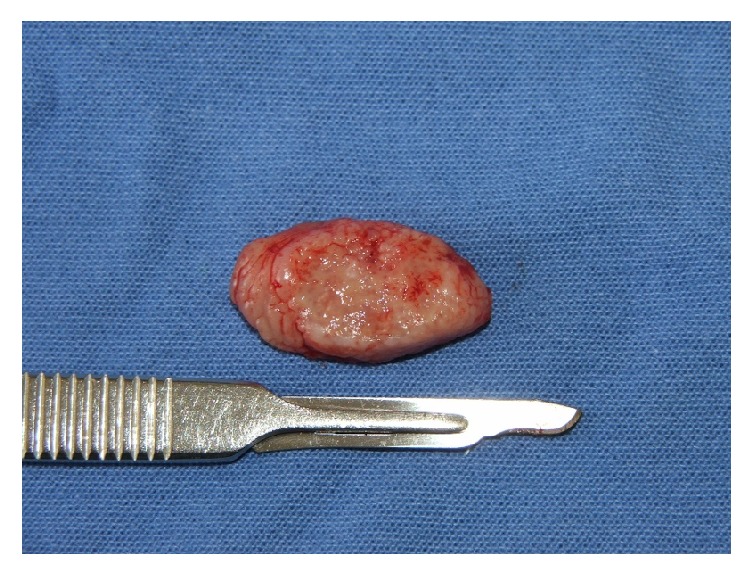
Excisional biopsy of the lesion.

A 60-year-old man from a rural area of Saltillo, northern Mexico, came to our institution presenting an ulcerated lesion on the left lateral border of the tongue of 1.5 cm of diameter. According to the patient the lesion has been growing for the last 5 months (Figure 1). He is a nonsmoker, with no evidences of any systemic disease. Considering the clinical aspects, age of the patient, and poor oral health, epidermoid carcinoma was considered as diagnosis. After an excisional biopsy the diagnosis was of coccidioidomycosis (Figure 2).

**Figure 3:**
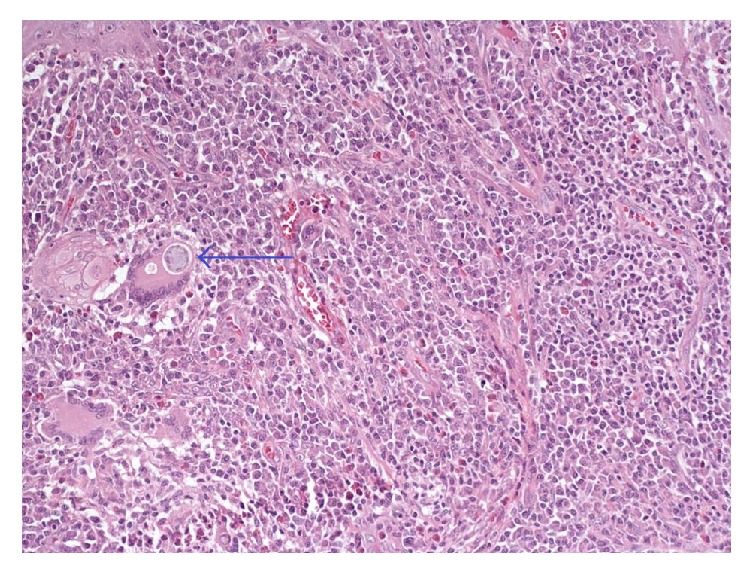
Chronic granulomatous inflammatory infiltrate, showing lymphocytes, plasma cells, and multinucleated giant cells containing spherical cytoplasmic inclusions corresponding to* Coccidioides immitis* (H&E; 200x).

**Figure 4:**
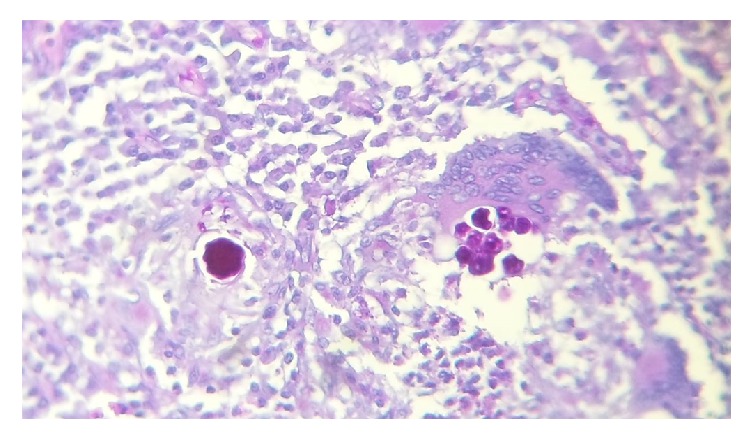
C. PAS staining highlighting various bodies of* C*.* immitis* in red color (PAS).

**Figure 5:**
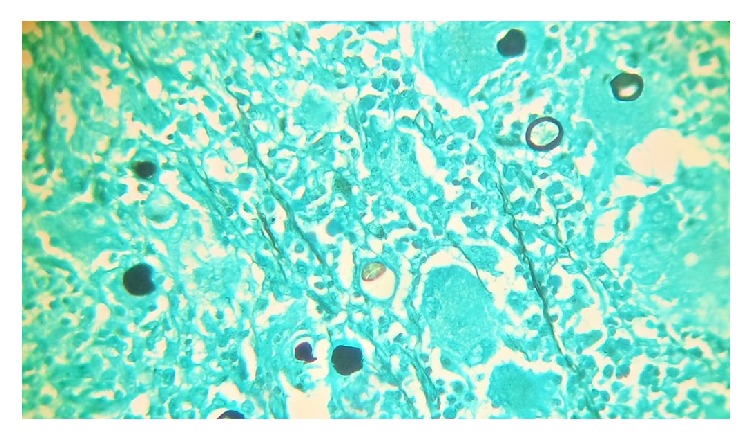
C. Grocott staining confirming the presence of large bodies of* C*.* immitis* in black (Grocott).

**Figure 6:**
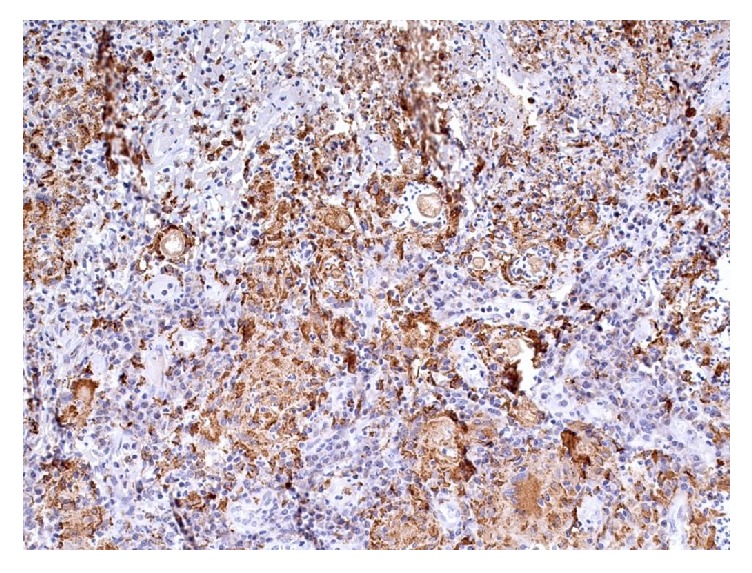
Immunohistochemistry showing granulomatous inflammation with predominance of macrophages and giant multinucleated cells positive for CD68, containing bodies of* C*.* immitis* (200x).

Histology demonstrated a granulomatous lesion with abundant lymphoplasmacytic chronic inflammatory infiltrate and multinucleated giant cells with spherical cytoplasmic inclusions of different size which correspond to the infectious agent* C. immitis* (Figures 3–6).

## 3. Differential Diagnosis

Ulcerated lesions of the mouth are common having variable causes; the most common are associated with trauma, infections, and epidermoid carcinoma, the latter usually presenting as a single ulcerated lesion in adults and elderly patients. Infections of the mouth causing ulcers include herpes simplex, syphilis, and depending on the immunological status of the patient and region of the world less common diseases should be considered particularly fungal infections as histoplasmosis, aspergillosis, cryptococcosis, paracoccidioidomycosis, and coccidioidomycosis. Many cases of tuberculosis have been found in the mouth and less commonly leishmaniasis and rarely hanseniasis. Early and correct diagnosis are essential for treatment and most of these diseases present systemic involvement [5, 6].

## 4. Treatment and Follow-Up

The patient was referred to the infectious disease department where supplementary studies were performed and systemic infection of coccidioidomycosis was confirmed and pulmonary lesions were evident on the chest X-ray. He received systemic antifungal therapy, itraconazole 200 mg tablets and 1 tablet by mouth per day during breakfast for three months, liver function tests were performed, and the results show no relevant data. Treatment was extended for another three months; we consider assessing liver function and tests were performed but this time the results were altered so it was decided to suspend the treatment for one month. Treatment was retaken for three months and by this time there was no evidence of the infection being eradicated completely; the treatment was extended for a year.

## 5. Discussion

Coccidioidomycosis is one of the deep mycoses that can be first diagnosed as an ulcer in the mouth [5, 6]. This infection is common in some areas, but it is important to consider the traveling history of the patient to endemic areas since some cases have been reported in places where this infection is rare [7]. Previous epidemiologic studies show that this mycosis is as prevalent in Mexico as in the endemic regions of the United States. The one carried out by González-Ochoa (National Survey 1961–1965) is the most important to date. Using the skin test with coccidioidin demonstrated variable infection rates in the states of Baja California, Chihuahua, Colima, Coahuila, Durango, Guanajuato, Guerrero, Jalisco, Michoacán, Nayarit, Nuevo León, San Luis Potosí, Sinaloa, Tamaulipas, and Zacatecas [8]. As far as Mexico is concerned the current impact of the disease is unknown but the increase of this mycosis in California and Arizona might have the same impact in Mexican nearby states. According to available information, more than 1,500 cases of primary coccidioidomycosis and 15 cases of disseminated disease are estimated annually in Mexico and some data point out that the prevalence of the disease decreased from west to east and from north to south [9]. There are two nearly identical species,* Coccidioides immitis* and* C*.* posadasii*, to determine the prevalence of these two species in northern Mexico; Bialek et al., using a polymerase chain reaction assay (LightCycler PCR), analyzed 120 strains isolated in clinical specimens during a 10-year period in Monterrey, Nuevo León. All strains studied corresponded to Silveira (now known as* C*.* posadasii*), an expected finding based on the geographical studies carried out by Fisher et al. [8] Although it has been investigated for years, there is as yet no clinically useful vaccine to prevent this disease. Therefore it is important for an accurate diagnosis and proper treatment a comprehensive clinical history and microscopical and complementary studies.
